# Montmorency cherry supplementation improves 15-km cycling time-trial performance

**DOI:** 10.1007/s00421-018-04058-6

**Published:** 2019-01-08

**Authors:** Paul T. Morgan, Matthew J. Barton, Joanna L. Bowtell

**Affiliations:** Department of Sport and Health Sciences, College of Life and Environmental Sciences, University of Exeter, St. Luke’s Campus, Heavitree Road, Exeter, EX1 2LU UK

**Keywords:** Flavonoids, Montmorency cherry, Oxidative stress, Polyphenols, Vascular function

## Abstract

**Aim:**

Montmorency cherries are rich in polyphenols that possess antioxidant, anti-inflammatory and vasoactive properties. We investigated whether 7-day Montmorency cherry powder supplementation improved cycling time-trial (TT) performance.

**Methods:**

8 trained male cyclists ($\dot {V}{{\text{O}}_{2{\text{peak}}}}$: 62.3 ± 10.1 ml kg^−1^ min^−1^) completed 10-min steady-state (SS) cycling at ~ 65% $\dot {V}{{\text{O}}_{2{\text{peak}}}}$ followed by a 15-km TT on two occasions. Participants consumed 6 pills per day (Montmorency cherry powder, MC; anthocyanin 257 mg day^−1^ or dextrose powder, PL) for a 7-day period, 3 pills in the morning and evening. Capillary blood [lactate] was measured at baseline, post SS and post TT. Pulmonary gas exchange and tissue oxygenation index (TOI) of *m*. vastus lateralis via near-infrared spectroscopy, were measured throughout.

**Results:**

TT completion time was 4.6 ± 2.9% faster following MC (1506 ± 86 s) supplementation compared to PL (1580 ± 102 s; *P* = 0.004). Blood [lactate] was significantly higher in MC after SS (PL: 4.4 ± 2.1 vs. MC: 6.7 ± 3.3 mM, *P* = 0.017) alongside an elevated baseline TOI (PL: 68.7 ± 2.1 vs. MC: 70.4 ± 2.3%, *P* = 0.018).

**Discussion:**

Montmorency cherry supplementation improved 15-km cycling TT performance. This improvement in exercise performance was accompanied by enhanced muscle oxygenation suggesting that the vasoactive properties of the Montmorency cherry polyphenols may underpin the ergogenic effects.

## Introduction

Reactive oxygen species (ROS) are continuously generated during repetitive muscular action from a variety of sources including enzymes such as NADPH oxidase and xanthine oxidase (Reid 2016a) in an intensity-dependent fashion (Bailey et al. 2007). Reactive oxygen species act as important signalling molecules and have been implicated in contraction-mediated increase in muscle glucose uptake (Merry and McConell 2009) and control of skeletal muscle blood flow (Trinity et al. 2016). It appears that under conditions of low oxidative stress and redox balance, ROS promote optimal vasodilation and hyperaemia in exercising muscle (Durand et al. 2015). However, under conditions of oxidative stress or already disturbed redox balance, ROS generation during exercise impairs blood flow and vasodilatory capacity (Donato et al. 2010).

Ryanodine receptors are the major calcium release channel in sarcoplasmic reticulum and due to the high number of cysteine residues in this protein, it is redox sensitive. Excess ROS generation has been shown to impair calcium handling and sensitivity, resulting in reduced contractile force development, thus impairing exercise performance (Reid 2016a). It is, therefore, plausible that elevated muscle antioxidant capacity may counteract fatigue and enhance performance during high intensity or prolonged exercise, by minimising disturbance of redox balance (Reid 2016b).

As a consequence, there is growing interest in the efficacy of fruit-derived polyphenol supplements, which possess antioxidant and anti-inflammatory properties, in improving exercise performance and/or tolerance both acutely (Cases et al. 2017; Deley et al. 2017; Oh et al. 2010; Trexler et al. 2014) and chronically (Braakhuis et al. 2014; Braakhuis and Hopkins 2015; Cook et al. 2015; Kang et al. 2012; MacRae and Mefferd 2006; Murphy et al. 2017; Sadowska-Krępa et al. 2008). Montmorency cherry polyphenols have been shown to enhance recovery of muscle strength following a bout of muscle-damaging exercise (Bowtell et al. 2011; Howatson et al. 2010). This enhanced exercise performance and functional recovery following muscle damage is likely to be mediated through the observed reduction in serum markers of oxidative damage after Montmorency cherry supplementation. These effects are suggested to be mediated via inhibition of superoxide producing enzymes such as NADPH oxidase or xanthine oxidase (Rodriguez-Mateos et al. 2013) or enhanced endogenous antioxidant capacity induced via nrf2 signalling (Huang et al. 2015). The resulting attenuation in superoxide exposure would reduce conversion of nitric oxide (NO) to peroxynitrite so preserving NO bioavailability during prolonged intense exercise (Benjamin et al. 1994; Cosby et al. 2003), and thus blood flow and tissue perfusion.

Polyphenol supplementation has also been implicated in increasing nitric oxide (NO) availability directly (Stoclet et al. 2004) by increasing the conversion of nitrite to NO (Rocha et al. 2009) as well as upregulating nitric oxide synthase (NOS, for review Galleano et al. 2010). Indeed, acute (Rodriguez-Mateos et al. 2013) and chronic (Khan et al. 2014) polyphenol supplementation has been linked to endothelium-dependent vasodilation. A meta-analysis found that supplementation with a mix of flavonoids increased flow-mediated dilatation (FMD) by 2.3% (based on 18 acute supplementation studies) and by 0.7% with chronic supplementation (based on 14 studies, Kay et al. 2012). This is likely to have a significant impact on exercise performance, during whole body exercise, where blood flow is considered to be a critical limiting factor to perfusion (Mortensen et al. 2008). Increased perfusion would result in increased tissue oxygen saturation and improved efflux of metabolic waste products such as lactate during exercise and subsequently enhance muscle function (Jacobs et al. 2011).

However, despite the potential for ergogenic effects of Montmorency cherry supplementation on exercise performance, very few studies have directly tested this hypothesis. Specifically, Montmorency cherry supplementation has recently been shown to enhance end-sprint cycling (Keane et al. 2018), aerobic running (Levers et al. 2016), and the recovery from prolonged, intermittent running performance (Bell et al. 2016) following a single, acute dose, of 7 or 8 days of supplementation, respectively.

The purpose of this study was to investigate the effect of 7-day Montmorency cherry supplementation on cycling time-trial (TT) performance. It was hypothesised that, compared to placebo, 7-day Montmorency cherry supplementation would: (1) enhance cycling TT performance, measured as a reduced time-to-complete the 15-km TT; (2) enhance tissue oxygenation during exercise, measured via the tissue oxygenation index (TOI); and (3) increase end-exercise capillary blood [lactate].

## Materials and methods

### Participants

Eight trained male competitive (> 250 miles/week) cyclists (mean ± SD: age: 19.7 ± 1.6 years, height: 1.79 ± 0.69 m, body mass: 75.0 ± 9.6 kg, $\dot {V}{{\text{O}}_{2{\text{peak}}}}$: 62.3 ± 10.1 ml kg^−1^ min^−1^, power output at $\dot {V}{{\text{O}}_{2{\text{peak}}}}$: 401 ± 38 W) volunteered and gave written informed consent to participate in this double blind crossover study, which had been approved by the University of Exeter Research Ethics Committee. A power analysis with an *α* error = 0.05, power = 0.95, and effect size = 2.89, was performed using the *G* × Power 3.1 analysis software (Heinrich Hein University, Duesseldorf, Germany), based on the effects of 7 days of blackcurrant supplementation on 16.1-km time-trial performance (Cook et al. 2015). This produced a minimum sample size of 4 participants. A total of eight participants were recruited to account for possible variation in the effects of Montmorency cherries and blackcurrants, and to maximise the statistical power for the secondary outcomes of TOI and other measures that may provide insight into the mechanisms of action. Participants reported to all testing sessions well-hydrated, having avoided strenuous exercise and caffeine ingestion for 24 and 3 h prior to testing, respectively. Participants were also instructed to consume their habitual diet and continue normal training activities for the first 5 days of the supplementation period but to refrain from strenuous physical activity for 48 h prior to the intensive exercise protocol. Participants recorded their diet and physical activity for 7 days prior to the cycling exercise trial (as described below) and then replicated this diet for the second, cross-over, trial. Testing was performed at the same time of day (± 2 h) for each subject.

### Experimental design

Participants visited the laboratory on 4 occasions during a 4-week period. All participants completed: (i) an incremental test to exhaustion; (ii) two familiarisation 15-km cycling TTs; and (iii) two 15-km cycling TTs following 7-day supplementation of placebo and Montmorency cherry supplementation. Experimental tests were randomised and counter-balanced, separated by a minimum 2-week wash-out period and performed on a customised TT bike (Planet X, Sheffield, UK) that replicated the set-up (namely seat post and handlebars) of their own competition bike to maximise ecological validity. The bike was then loaded onto a static trainer to complete TT simulations within the lab (Kinetic Magnetic, Minneapolis, USA). Power output and work done (and distance) were measured via a mobile power meter integrated into the rear wheel (PowerTap G3 Hub, Madison, USA) connected wirelessly to a data logger (PowerTap Joule, Madison, WI, USA). The PowerTap G3 device was zeroed before each test. All laboratory-based tests were performed in similar environmental conditions (temperature, 18–20 °C; relative humidity, 45–55%). Participants were provided with feedback regarding the elapsed work done and distance completed as well as the work and distance remaining during the laboratory TTs at 5-km intervals.

### Incremental test

On the first laboratory visit, participants performed a ramp-incremental cycling test for the determination of peak oxygen uptake ($\dot {V}{{\text{O}}_{2{\text{peak}}}}$) and calculation of a heavy-intensity work load for steady-state (SS) exercise on an electronically braked cycle ergometer (Corival, Lode BV, Groningen, The Netherlands). The test was preceded by 3 min of ‘baseline’ cycling at 20 W after which the workload increased to 75 W. The work load then continued to increase at a rate of 30 W min^−1^ until the limit of exercise tolerance. Participants pedalled at a self-selected cadence between 80 and 100 rpm. The test was terminated when cadence dropped by more than 10 rpm from the selected cadence for more than 10 s despite strong verbal encouragement. The $\dot {V}{{\text{O}}_{2{\text{peak}}}}$ was calculated as the highest 30-s average value attained before volitional exhaustion.

### Experimental trial

Following familiarisation, participants completed two trials under the experimental conditions, placebo (PL) and Montmorency cherry supplementation (MC). To assess differences in muscle tissue oxygenation, initially, participants completed 3 min of baseline cycling (i.e., 20 W) before an abrupt increase to the work rate which corresponded to ~ 65% $\dot {V}{{\text{O}}_{2{\text{peak}}}}$ (steady-state exercise) for 10 min on a lode cycle ergometer. Following a 5-min period of rest, participants then completed a 15-km TT in the shortest time possible on a TT bike (as described above). Muscle tissue oxygen saturation, blood lactate (BLa) and pulmonary gas exchange (detailed below) were measured throughout.

### Muscle oxygenation

**Fig. 1:**
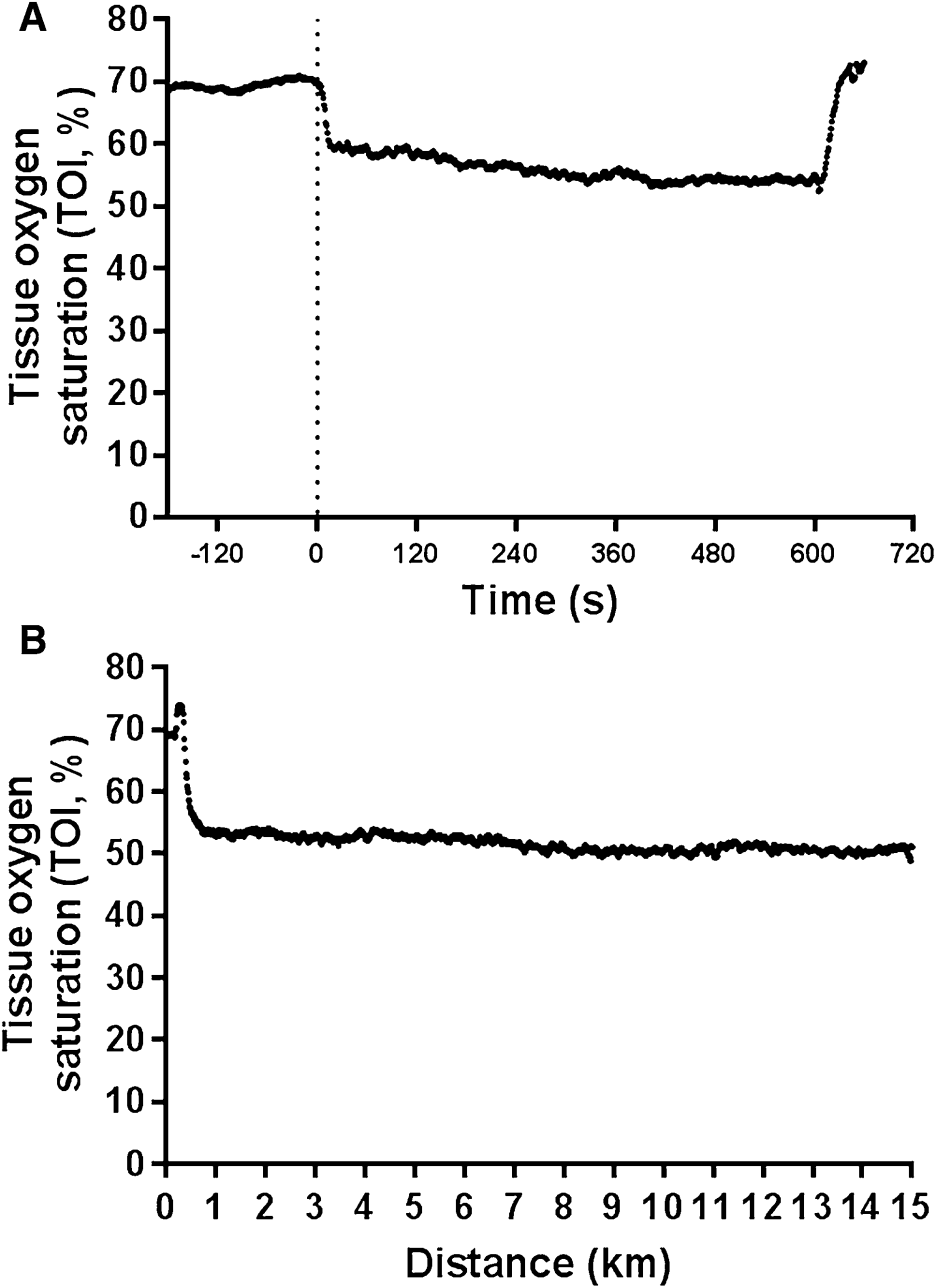
Exemplar plot of TOI during steady-state (**a**) and time-trial (**b**) exercise. Tissue oxygenation index (i.e., TOI) was calculated as the percentage of total haemoglobin and myoglobin that was oxygenated. During steady-state exercise, TOI was averaged across the final 5 min of the 10-min bout. TOI was averaged in three work periods corresponding to 5-km intervals during TT exercise

For all experimental visits, muscle oxygenation status of the *m*. vastus lateralis of the right leg was monitored using near-infrared spectroscopy (Portamon, Artinis medical systems, The Netherlands). The CV of the device in our lab during unloaded cycling was recorded at 1.3 ± 0.5%. On arrival at the laboratory, the skin area underneath the near-infrared spectroscopy device was shaved, then exfoliated and cleaned with alcohol to minimise skin impedance. The sensor was placed at the midpoint between the lateral epicondyle of the femur and the femoral head. Adhesive tape and a hypoallergenic medical tape were used to ensure the sensor stability. An elastic bandage was wrapped around the participants’ leg and secured with adhesive tape to ensure the sensor did not move during exercise as well as minimising potential of extraneous light influencing the signal. The transmitted light was recorded at 10 Hz but down-sampled and exported at 1 Hz into proprietary software (Oxysoft, Artinis medical systems, The Netherlands). Tissue oxygenation index (i.e., TOI) was calculated as the percentage of total haemoglobin and myoglobin that was oxygenated. During SS exercise, TOI was averaged across the final 5 min of the 10-min bout. TOI was averaged in three work periods corresponding to 5-km intervals during TT exercise (i.e., 0–5 , 5–10 , 10–15 km) (Fig. 1).

### Measurements

Throughout all tests, pulmonary gas exchange and ventilation was measured using an online gas analyser (Cortex Metalyzer 2R, Leipzig, Germany) with a CV of < 2% (Macfarlane and Wong 2012). A volume transducer was securely attached to a facemask and a capillary line was connected to the mask allowing inspired and expired gas volume and gas concentration to be collected. Known gas concentrations were used to calibrate gas analysers in line with manufacturer’s recommendations. The turbine volume transducer was calibrated using a 3-L syringe (Hans Rudolph, Kansas City, MO). Fingertip capillary blood samples were collected at baseline, post SS cycling and immediately after the completion of TT for the assessment of BLa using a portable lactate analyser (Lactate Pro, Arkray Inc, Japan). The CV using this device has been shown to be < 3.6% (Bonaventura et al. 2015).

### Nutritional supplements

Each cherry capsule (CherryActive® Ltd, MC) consisted of finely powdered freeze-dried Montmorency cherry surrounded by a gelatine shell containing 0.3 g CHO and provided a total of 1.3 kCal. Tart cherry powder was analysed for total polyphenol content (Folin-Denis method) and anthocyanin content (high performance liquid chromatography, Atlas Bioscience Inc., Tucson, AZ, USA). Total polyphenol content provided by the 6 capsules, consumed daily (3 in the morning and 3 in the evening), was 462.8 mg day^−1^ and total anthocyanin content was 256.8 mg day^−1^. On day 7 (day of experimental testing), participants consumed 3 pills 60 min prior to the first experimental test. The optimal dose identified in a recent meta-analysis (Kay et al. 2012) was 500 mg per day total flavonoids or 300 mg per day of procyanidins, and the dose provided in the present study was based on this work. The placebo was made from dextrose powder inserted into gelatine capsules designed to have a similar appearance to cherry capsules but without the phytochemical content. To ensure compliance, in addition to recording diet and physical activity, participants were required to complete a 7-day supplementation record. This was then repeated in the crossover trial. For all participants, compliance was recorded at 100%.

### Statistical analysis

Paired samples *t* test was used to compare mean $\dot {V}{{\text{O}}_2}$, respiratory exchange ratio and TOI for the final 5 min of SS exercise between PL and MC. In addition, paired samples *t* test was used to assess the differences in completion times of the 15-km TT between conditions. Data normality assumptions were assessed using Kolmogorov–Smirnov test. Pearson’s product–moment correlation was used to examine the inter-relationship between TOI and the percentage of $\dot {V}{{\text{O}}_{2{\text{peak}}}}$ during SS exercise. Specifically, the correlation between the difference in TOI between conditions and the percentage of $\dot {V}{{\text{O}}_{2{\text{peak}}}}$ during SS exercise was calculated. Profiles of TT performance split times, $\dot {V}{{\text{O}}_2}$ and TOI were analysed using two-way ANOVAs with repeated measures (condition [placebo vs. MC] × distance: [first, middle and final 5-km averages]). BLa was analysed using a two-way repeated-measures ANOVA with two timepoints (baseline and end-exercise). Where the ANOVA revealed a significant interaction effect, a post hoc *t* test was conducted using a Bonferroni correction. For calculation of effect size, partial eta squared (*η*^2^) was used for omnibus tests and Cohen’s *d* was used to calculate the effect size for paired *t* tests. In addition, Cohen’s *d* was corrected for the paired *t* test for dependence. Where sphericity was violated, a Greenhouse-Geisser correction factor was used. For all tests, results were considered statistically significant when *P* < 0.05. Data are presented as means ± SD unless otherwise indicated. All statistical analyses were conducted using IBM SPSS Statistics version 24.

## Results

### Physiological responses to steady-state (SS) exercise

**Fig. 2:**
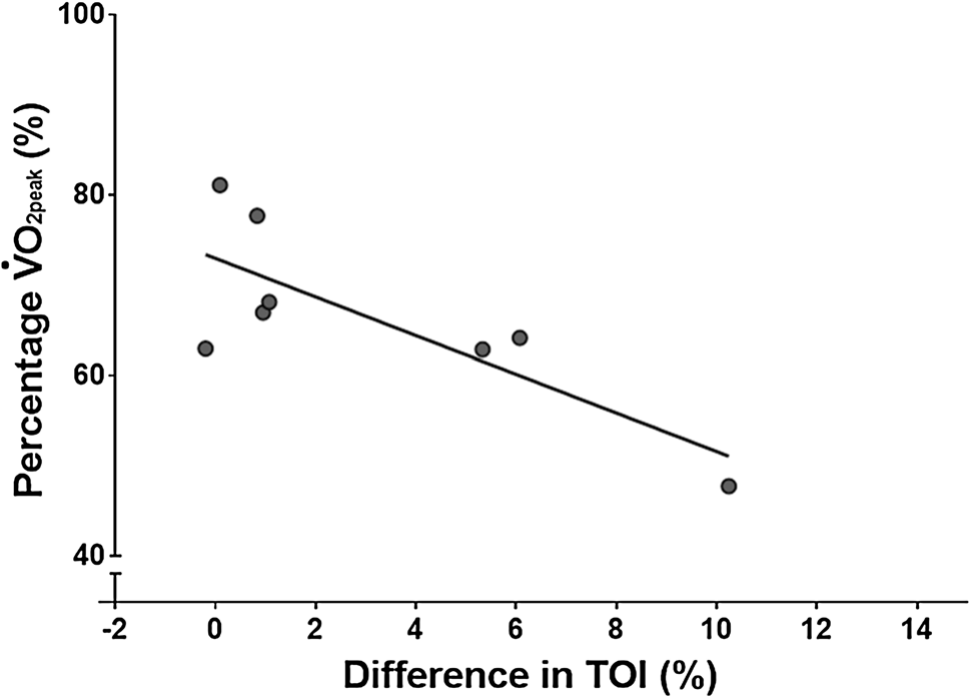
Difference in tissue oxygenation index (TOI) between cherry and placebo trials during steady-state exercise for each participant. A significant correlation was found between the difference in TOI between trials and SS percentage of $\dot {V}{{\text{O}}_{2{\text{peak}}}}$ (*r* = − 0.79, *r*^2^ = 0.62, *P* < 0.05) with the cherry supplementation shown to yield larger changes in TOI compared to placebo trial at lower relative $\dot {V}{{\text{O}}_{\text{2}}}$

The work rate required to elicit ~ 65% $\dot {V}{{\text{O}}_{2{\text{peak}}}}$ during SS exercise, determined during the preliminary incremental test, was 235 ± 38 W, and 141 ± 23 kJ of work was completed during SS exercise. The physiological responses to SS exercise for MC and PL can be seen in Table 1. Baseline TOI was significantly higher in MC (70.4 ± 2.3%) compared to PL (68.7 ± 2.1%, *P* = 0.02, *d* = 0.76, Table 1). In addition, whilst there was not a statistically significant difference in mean TOI during SS exercise following MC supplementation (57.4 ± 1.7 vs. 54.4 ± 6.9%, *P* = 0.06, *d* = 0.52), the difference in TOI between PL and MC trials was negatively correlated with SS relative exercise intensity (i.e., percentage of $\dot {V}{{\text{O}}_{2{\text{peak}}}}$, *r* = − 0.79, *P* = 0.02, Fig. 2). Baseline BLa was similar between trials (PL: 1.7 ± 0.4, MC: 1.5 ± 0.4 mM, *P* = 0.31, *d* = 0.38 Table 1). However, end-exercise BLa was significantly higher in MC (PL: 4.4 ± 2.1, MC: 6.7 ± 3.3 mM, *P* = 0.02, *d* = 0.86, Table 1). There was no difference in mean V̇O_2_ (PL: 3.1 ± 0.5 vs. MC: 3.1 ± 0.6 l min^−1^, *P* = 0.23, *d* = 0.10) or respiratory exchange ratio (PL: 0.96 ± 0.06, MC: 0.93 ± 0.07, *P* = 0.19, *d* = 0.49) between conditions.

**Table 1:** Physiological responses to steady-state and 15-km cycling TT

	Placebo (PL)	Cherry Active (MC)
Steady-state exercise		
End-exercise lactate (mmol/L)	4.4 ± 2.1	6.7 ± 3.3^a^
End-exercise RER	0.96 ± 0.06	0.93 ± 0.07
Baseline TOI (%)	68.7 ± 2.1	70.4 ± 2.3*
Mean SS exercise TOI (%)	54.4 ± 6.8	57.4 ± 4.8
Mean $\dot {V}{{\text{O}}_{\text{2}}}$ (L/min)	3.1 ± 0.5	3.1 ± 0.6
15-km time-trial		
TT performance (s)	1580 ± 102	1506 ± 86*
End-exercise lactate (mmol/L)	12.4 ± 3.9	12.1 ± 4.2
End-exercise RER	0.99 ± 0.05	0.96 ± 0.06
End-exercise TOI (%)	53.7 ± 7.2	55.1 ± 7.7
Mean $\dot {V}{{\text{O}}_{\text{2}}}$ (L/min)	3.4 ± 0.6	3.5 ± 0.5

*RER* respiratory exchange ratio,* TOI* tissue oxygen saturation index,* TT* time-trial

^a^Significantly different from PL, *P* < 0.05

### 15-km cycling TT performance

**Fig. 3:**
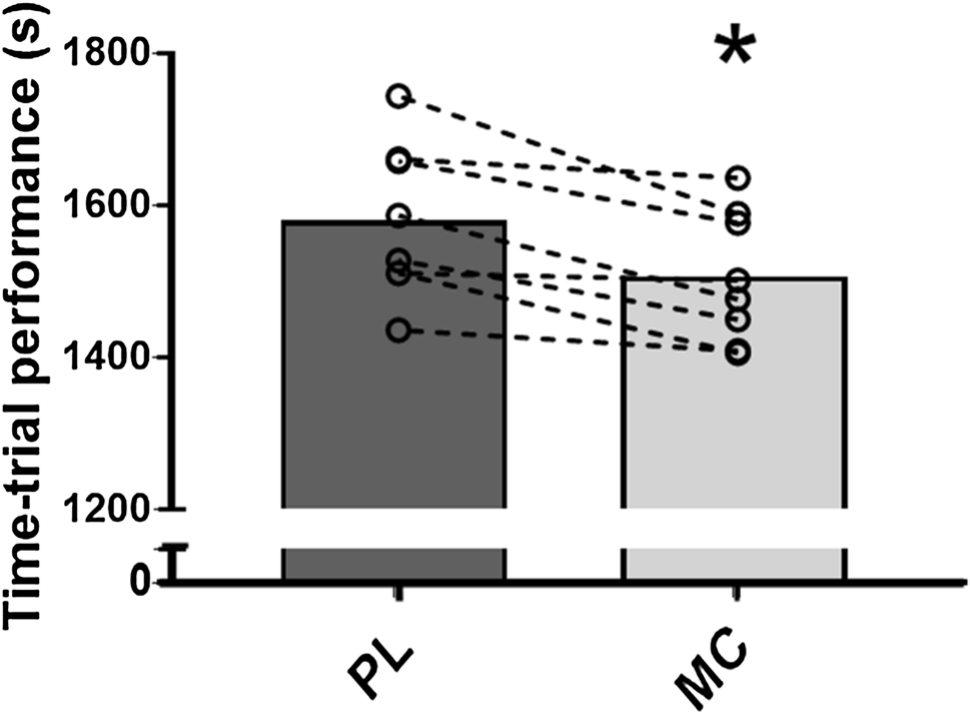
Mean completion time for the 15-km TT following placebo (dark grey) and cherry supplementation (grey). Completion time was significantly decreased following cherry supplementation (*P* < 0.05). Of the 8 participants, all 8 completed the TT in a quicker time following cherry supplementation compared to placebo, ranging from a 9 s (0.6%) improvement to a 155 s (8.9%) improvement. Asterisk significantly different to placebo (*P* < 0.05)

**Fig. 4:**
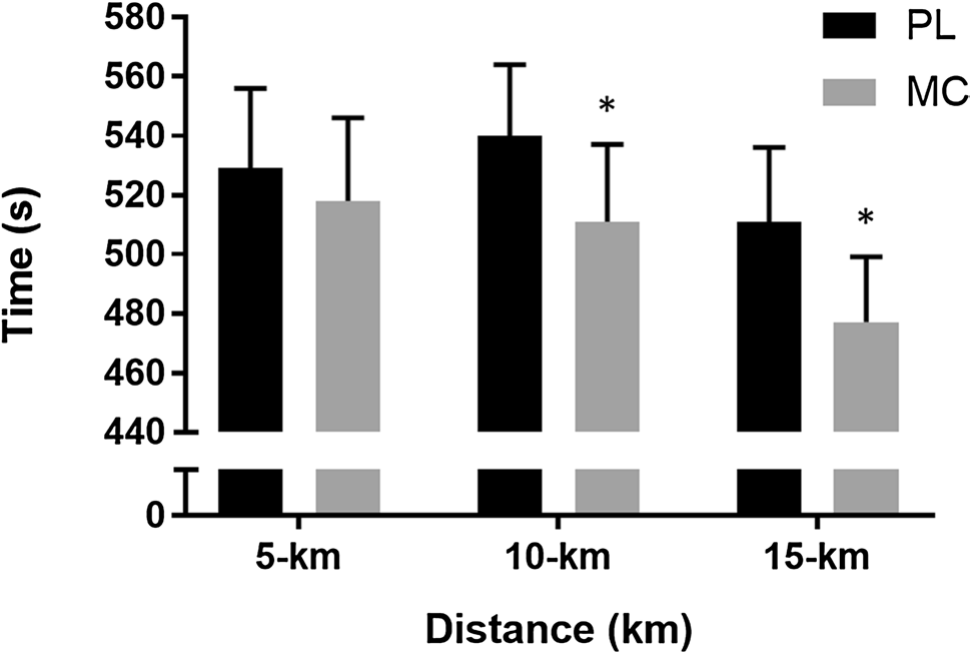
Mean ± SD completion time for the 15-km TT following placebo (dark grey) and cherry supplementation (grey) in 5-km intervals. Completion time was significantly decreased following cherry supplementation (*P* < 0.05). TT time was significantly different at 10- and 15-km time points. Asterisk significantly different to placebo (*P* < 0.05)

Mean completion time for the 15-km TT was 4.6 ± 2.9% (74 ± 50 s) faster in MC (1506 ± 86 s) compared to PL (1580 ± 102 s, *P* < 0.01, *d* = 0.78, Fig. 3). In addition, there was a significant interaction effect (distance × condition, *P* < 0.0001, *η*^2^ = 0.75) and the main effects of distance (*P* < 0.0001, *η*^2^ = 0.99) and condition (*P* = 0.02, *η*^2^ = 0.58) on time taken to complete each 5 km block of the TT, indicating improved performance following MC supplementation. Post hoc *t* test confirmed that there was a difference between conditions during the middle (i.e., 5–10 km) and final 5-km intervals (all *P* < 0.0001, Fig. 4). This improvement in mean group performance was consistent in all 8 participants, ranging from a 0.6 to 8.9% reduction in time to complete the 15-km TT (Fig. 3).

### 15-km cycling physiological responses

BLa was significantly increased after the TT (*P* < 0.0001, *η*^2^ = 0.90, Table 1). However, no interaction effect or main effect of condition were observed between MC (12.1 ± 4.2 mM) and PL (12.4 ± 3.9 mM, *P* = 0.82) immediately post TT. There was also no interaction effect or main effect of condition on TOI between MC (55.1 ± 7.7%) and PL (53.7 ± 7.2%, *P* = 0.70), despite observing a significant decrease in TOI during the TT (main time effect: *P* < 0.0001, *η*^2^ = 0.71). Similarly, analysis of the V̇O_2_ profile revealed no interaction effect or main effect of condition (mean $\dot {V}{{\text{O}}_{\text{2}}}$ PL: 3.4 ± 0.6 l min^−1^ MC: 3.5 ± 0.5 l min^−1^*P* = 0.70). A main effect of time on $\dot {V}{{\text{O}}_{\text{2}}}$ was observed during the TT (main time effect: *P* < 0.0001, *η*^2^ = 0.74), reflecting increase in oxygen consumption as the TT progressed. Asterisk significantly different from placebo (*P* < 0.05)

## Discussion

The main novel finding of this study was that, consistent with our primary hypothesis, 15-km TT performance was improved following 7-day MC supplementation compared with PL in a group of trained male cyclists. This improvement in exercise performance was accompanied by a significant increase in resting (‘baseline’) *m*. vastus lateralis oxygenation.

In the current study, 15-km cycling TT performance was improved by ~ 4.6% following MC relative to PL. MC supplementation has been shown to decrease markers of inflammation and oxidative stress (Bell et al. 2014), as well as enhance recovery from muscle damage (Bowtell et al. 2011; Howatson et al. 2010). The magnitude of improvement in TT performance in our study is larger than that proposed as the “smallest worthwhile change” for road TT cycling (Paton and Hopkins 2006) and are similar to acute nitrate supplementation of similar TT duration (Lansley et al. 2011). However, it is pertinent to note that whilst the magnitude of improvement in mean group TT performance was larger than that proposed as the smallest worthwhile change, only 6 of 8 subjects experienced improvements in TT performance that exceeded the smallest worthwhile change. Thus, whilst all 8 subjects experienced an ‘improvement’ in TT performance, the ‘differences’ observed within 2 participants were within the day-to-day variability of the measurement. As a consequence, we only observed a medium effect size of supplementation on TT performance (*d* = 0.78).

However, our findings support recent observations on the potential of MC supplementation to improve exercise performance (Keane et al. 2018; Levers et al. 2016). Specifically, Keane et al. (2018) found a single acute dose of Montmorency cherry concentrate (60 ml, providing 73 mg cyanidin-3-glucoside per L) to increase peak power by 9.5% in trained endurance cyclists. Whereas, Levers et al. (2016), demonstrated a freeze-dried Montmorency cherry supplement (480 mg daily, ~ 990 mg polyphenols including ~ 66 mg anthocyanins, 7 days before, the day of, and 2 days after completing a half-marathon) to attenuate post-run markers of muscle catabolism and physiological stress (dampening of the inflammatory response and better maintenance of redox balance) in trained individuals. However, it should be noted that this study randomised participants into independent groups matched for reported race pace and thus did not perform a crossover design. In addition, a ‘non-significant trend’ (*P* = 0.09) was observed for an improvement in running performance (~ 13%) when compared to predicted race pace. In addition, many studies, including Keane et al. (2018), ask participants to adhere to a restricted diet in the days preceding the trials, highlighting the need for further work in investigating the potential synergetic effects of MC supplementation within habitual dietary practices. This is particularly important for athletes who typically have a diet high in polyphenols.

Our findings are in agreement with Cook et al. (2015) who reported enhanced 16.1-km cycling TT performance by ~ 2.4% following 7-day blackcurrant supplementation (providing 105 mg day^−1^ anthocyanins). A number of other studies have also observed enhanced exercise performance after acute polyphenol supplementation during, for example, treadmill running (ecklonia cava, Oh et al. 2010) and repeated all-out cycling (grape, pomegranate and green tea blend, Cases et al. 2017; pomegranate extract; Trexler et al. 2014). Chronic (7 day) blackcurrant polyphenol (105 mg anthocyanin, Cook et al. 2015; Perkins et al. 2015; Murphy et al. 2017) and 2-day epigallocatechin (EGCG, Richards et al. 2010) supplementation have also been shown to enhance exercise performance.

In contrast, a previous study has reported no improvement in 20-km TT performance in moderately trained individuals following MC supplementation despite a 3-day ingestion of dried Montmorency cherries which provided 216 mg of polyphenol of which the final dose was administered 2–3 h prior to the cycling trial (Clifford et al. 2013). In addition, not all studies have found ergogenic effects of polyphenol ingestion (Crum et al. 2017; Labonté et al. 2013). The absence of effect in these studies may relate to differences in dosing (i.e., 462 vs. 216 mg; 7 vs. 3 days, Clifford et al. 2013), training status (i.e., novice vs. athlete, Crum et al. 2017; Labonté et al. 2013), environmental conditions (i.e., high altitude, Crum et al. 2017), current antioxidant status (i.e., high vs. low, Green et al. 2004) as well as the timing of intake (i.e., 60 min vs. 2–3 h prior to exercise, Clifford et al. 2013) which may have marked effects on the ergogenic effects of polyphenol supplementation. The current investigation used MC powdered capsules in trained cyclists, using a TT scenario and a high polyphenol dose (462 mg polyphenols day^−1^) for 7 days, combined with a maintenance of habitual diet (i.e., with no polyphenol restrictions), thereby potentially explaining some of the differences observed between studies and maximising the ecological validity of our findings, respectively. In addition, the last supplement dose on day 7 was taken 60 min prior to exercise, since it has been observed that the endothelial-dependent vasodilatation response peaks approximately 60 min after ingestion of blueberry polyphenols (Rodriguez-Mateos et al. 2013). However, other studies have timed the polyphenol dose to coincide with peak plasma anthocyanin metabolite concentration which occurs 90–120 min following consumption (Keane et al. 2018).

We also observed a significant increase in baseline muscle oxygenation (i.e., TOI) with a medium effect size (*d* = 0.76), suggesting increased perfusion following MC supplementation. However, there was no statistically significant effect of MC on TOI during SS exercise (d = 0.52), which may indicate that the study was underpowered to detect an effect on TOI during exercise. There was a significant negative correlation in the difference in TOI between conditions with the percentage of $\dot {V}{{\text{O}}_{2{\text{peak}}}}$ suggesting that the increase in TOI after MC supplementation was more pronounced at lower exercise intensities. In agreement with this observation of increased perfusion with MC supplementation, enhanced vascular function has been implicated in other studies following polyphenol supplementation, which paralleled the ergogenic effects for performance (blackcurrant, Cook et al. 2015; pomegranate; Roelofs et al. 2017; pomegranate; Trexler et al. 2014). This suggestion of increased perfusion is corroborated by the elevated BLa in the cherry trial, which is unlikely to be due to increased lactate production, since oxygen consumption and the amount of work completed during 10-min SS cycle exercise at 65% $\dot {V}{{\text{O}}_{2{\text{peak}}}}$ were identical between trials. Rather, the elevated BLa is most likely due to increased lactate efflux from the muscle as a consequence of higher perfusion (Richardson et al. 1998). These observations are consistent with previous findings of reduced blood pressure following MC supplementation (Keane et al. 2018), providing further support that the enhanced performance might be mediated through the vasodilatory properties of polyphenol-rich MC.

These vasoactive properties of fruit-derived polyphenols have been also been demonstrated in resting conditions, with enhanced FMD evident after acute blueberry (Rodriguez-Mateos et al. 2013) and chronic blackcurrant (Khan et al. 2014) supplementation. The improvement in FMD is, by definition, a result of increased NO bioavailability, since FMD is NO dependent (Pyke and Tschakovsky 2005). It is likely that similar mechanisms underpin the enhanced muscle oxygenation observed in the present study and the improved arterio-venous difference postulated by others (Richards et al. 2010). However, Keane et al. (2018) recently demonstrated an improvement in end-sprint cycling performance despite no differences in plasma nitrite concentration between MC and PL suggesting that the improvements in performance with MC supplementation appear to be independent of NO-mediated signalling (and thus perfusion) and likely due to a reduction in ROS production. Although plasma nitrite may not be a sufficiently sensitive measure of NO in muscle (see Bryan and Grisham 2007 for review). However, our results do corroborate Keane et al. (2018) as, during all-out cycling where the participant has an ability to manipulate work output, we observed no differences in TOI.

During high-intensity exercise, excessive ROS production can lead to cellular damage and oxidative stress (Powers et al. 2004; MacRae and Mefferd 2006). There is evidence that acute (dark chocolate, Davison et al. 2012; black grape, raspberry and redcurrant polyphenol blend; Morillas-Ruiz et al. 2006; curcumin; Takahashi et al. 2014) and chronic (blueberry, McAnulty et al. 2011) polyphenol supplementation protects against endurance exercise-induced oxidative damage, but unfortunately these studies did not include an assessment of exercise performance. Whereas in the present study, whilst performance was enhanced, no measures of oxidative damage are available. However, MC concentrate has been shown to reduce oxidative damage after intense exercise (i.e., Bowtell et al. 2011; Howatson et al. 2010).

Previous literature has reported that the baseline antioxidant profile of an individual is an important determinant of the ergogenic effectiveness of an antioxidant intervention (Paschalis et al. 2018). In contrast to some other studies, no dietary restrictions to reduce polyphenol intake were imposed in the present study and ergogenic effects were nonetheless evident. The improvement in cycling performance in the current study could prove beneficial in elite sporting performance where athletes are attempting to find small but significant improvements in performance. Our findings are especially pertinent for highly trained individuals who demonstrate optimal NOS expression and high habitual dietary intakes of antioxidants to combat oxidative stress (Green et al. 2004).

In conclusion, 7-day Montmorency cherry powder supplementation enhanced 15-km cycling TT performance. This improvement in exercise performance seems to involve enhanced muscle perfusion as evidenced by increased muscle oxygenation presumably due to the vasoactive and anti-oxidative effects of the phytochemicals within the Montmorency cherries. The results of this study suggest that supplementation with MC concentrate might represent, a practical, non-pharmacological, dietary intervention to reduce enhance cycling performance in trained individuals. However, further research is required to investigate the dose–response between MC supplementation and cycling performance as well as the precise mechanisms responsible for this ergogenic potential, especially in the presence of a diet high in polyphenols.

## Abbreviations

BLa: Blood lactate
CV: Coefficient of variation
FMD: Flow-mediated dilatation
MC: Montmorency cherry
NO: Nitric oxide
PL: Placebo
ROS: Reactive oxygen species
SS: Steady-state exercise
TOI: Tissue oxygenation index
TT: Time-trial
$\dot {V}{{\text{O}}_{2{\text{peak}}}}$: Peak oxygen uptake
